# The Classification, Diagnosis and Prognosis of Tumors

**Published:** 1861-07

**Authors:** 


THE CLASSIFICATION, DIAGNOSIS AND PROGNOSIS OF TUMORS.—Briefly delineated for Practitioners by Dr. THEODORE BILLROTH, (now Professor of Surgery at the University of Zurich ) From the Deutsche Klinik, 1859. Translated from the German by G. BAUMGARTEN, M. D. St. Louis, Mo. 1861.

The thanks of the profession are due Dr BAUMGARTEN, for his very faithful translation of this interesting monograph, a summary of which we may soon give in our pages. The leading idea of Prof. Billroth's paper is that all systems of classification of pseudo-plasms which rely solely on microscopical and chemical analysis are deceptive, because, while it is true, in normal histology, that *like structure occasions like function*, it is equally true of pathological tissues, that *tumors, whose tissues are identical, often have a very different clinical significance;* again, that classifications based on modes of development,—or on the manner in which the new tissues are inserted into the old are also fallacious; the division into tumors which do and do not recur after extirpation, impracticable and untenable; while the contemplation of every histologically defined form of tumor with regard to its connection with different organs and in the light of clinical experiene, is cumbersome and complicated. A consideration and investigation of all these culminates in the dictum that in the classification of pseudo-plasms *the histological consideration must combine with the clinical, but the latter must be the leading principle.*

Prof. B. is by no means an ultraist in his views, although his contempt for young microscopists verges on the extremes of politeness sometimes. While anxious to advance, he fails not to keep in sight what has already been accomplished; and his efforts look to a union of what is worthy in the old, with the proved improvements of the new, *e. g.:*

The prognostic principle of classification, which hitherto was familiar to physicians—the faculty of recurrence in varible degree and extent—I regard as perfectly competent for practical purposes, and therefore retain it in the main. In the designation of the various forms of tumors I have likewise altered nothing and retained the usual names, but eliminated those names, which were chosen according to microscopical elements; they may be reserved to histologists for the more minute distinctions. The consistency and the similarity to normal forms have occasioned the more current names, and it would be a vain endeavor to substitute other denominations for them; the majority have been chosen very pertinently by our forefathers.

In closing he adds:

The classification of tumors herewith concluded, has, like every essay of this kind, its great imperfections, I well know; but as the above synopsis corresponds to observation and to practical wants, as I hope, it thereby accomplishes that purpose, which every classification of diseases can only have, namely, to facilitate the mutual understanding of colleagues. Many will reject the principle of classification; I am aware myself, that much can be said against it; but that for practical medicine, without detriment to science, the practical points of view, *i. e.*, those derived directly from clinical experience, the observation at the bedside, must always be placed in the foreground,—is a principle the physician should never lose sight of. I believe, that it will favor the popularity of the four groups advanced by me, to give each group a general name. I have no new names to suggest, but only propose to apply the old names in the manner they are used in the following synoptical table, with reference to the above detailed remarks:

I. Benign Tumors; *i. e., such as but seldom return after extirpation, but sometimes occur distributed in great numbers over the whole surface of the body.*

1. The simple Cysts. (*a*) With serous fluid. (*b*) With mucous contents. (*c*) With pultaceous contents. (*d*) With blood. 2. The Fatty Tumors. 3. The Fibrous Tumors. (*a*) The soft fibrous tumors. (*b*) The hard fibrous tumors. 4. The pure Cartilaginous Tumors. 5. The Exostoses. (*a*) The spongy exostoses. (*b*) The ivory exostoses. 6. The Vascular Tumors. (*a*) The telangiectases. (*b*) The cavernous hæmatomata. 7. The Horny Excrescences.

II. Sarcomata; *Tumors which often return locally, but seldom invade the internal organs.*

1. The Gland-like Tumors. 2. The Colloid Tumors. (*a*) The homogeneous colloid sarcomata. (*b*) The areolar colloid tumors. 3. The Cystoids and Cystosarcomata. 4. The firm Sarcomata. 5. The soft Sarcomata. 6. The soft Papillary Tumors.

III. Carcinomatous Tumors; *i. e., such as always return locally, then appear in the nearest lymphatic glands, and finally in internal organs.*

1. The Carcinomata. 2. The Cancroids. 3. The Scirrhi.

IV. Medullary and Melanotic Tumors; *i. e., such as usually soon return locally, and rapidly extend upon internal organs.*

1. The Medullary Fungi. 2. The Melanotic Tumors.

